# Numerical Optimization of an Open-Ended Coaxial Slot Applicator for the Detection and Microwave Ablation of Tumors

**DOI:** 10.3390/biology10090914

**Published:** 2021-09-14

**Authors:** Carolin Hessinger, Martin Schüßler, Sabrina Klos, Markus Kochanek, Rolf Jakoby

**Affiliations:** 1 Institute for Microwave Engineering and Photonics, Technische Universität Darmstadt, Merckstr. 25, 64283 Darmstadt, Germany; martin.schuessler@tu-darmstadt.de (M.S.); markus.kochanek@tu-darmstadt.de (M.K.); rolf.jakoby@tu-darmstadt.de (R.J.); 2 Communications Engineering Lab, Technische Universität Darmstadt, 64289 Darmstadt, Germany; sabrina.klos@tu-darmstadt.de

**Keywords:** microwave ablation, microwave applicator, multiobjective optimization

## Abstract

**Simple Summary:**

In this work, a parametric analysis of a dual-mode applicator for microwave ablation treatments is proposed to optimize the geometric parameters of the structure. The dual-mode concept means that the applicator comprises an additional sensing mode to determine the dielectric properties of the surrounding tissue before and during the ablation procedure. Based on numerical electromagnetic-thermal coupled simulations the most optimal design in terms of applicator efficiency as well as ablation zone volume and shape is determined that further fulfills the sensitivity requirements of the sensing mode. The multiobjective optimization problem is solved graphically with the so-called Pareto-optimization method. The resulting Pareto-optimal dual-mode applicator designs are characterized by electromagnetic and thermal simulations and discussed.

**Abstract:**

A multiobjective optimization method for a dual-mode microwave applicator is proposed. Dual-modality means that microwaves are used apart from the treatment, and also for the monitoring of the microwave ablation intervention. (1) The use of computational models to develop and improve microwave ablation applicator geometries is essential for further advances in this field. (2) Numerical electromagnetic–thermal coupled simulation models are used to analyze the performance of the dual-mode applicator in liver tissue; the sensitivity evaluation of the dual-mode applicator’s sensing mode constrains the set of optimal solutions. (3) Three Pareto-optimal design parameter sets are derived that are optimal in terms of applicator efficiency as well as volume and sphericity of the ablation zone. The resulting designs of the dual-mode applicator provide a suitable sensitivity to distinguish between healthy and tumorous liver tissue. (4) The optimized designs are presented and numerically characterized. An improvement on the performance of previously proposed dual-mode applicator designs is achieved. The multiphysical simulation model of electromagnetic and thermal properties of the applicator is applicable for future comprehensive design procedures.

## 1. Introduction

Liver cancer is the sixth most frequently diagnosed cancer worldwide, with an increasing incidence rate [1]. Thanks to intensive research in the field of therapy of oncological diseases, its prevalence has increased in recent years. Classical therapy methods include surgery, radiation, and chemotherapy with the aim of completely destroying the tumor. Newer therapies include immunotherapy, and personalized and local therapies, such as thermal ablation procedures that use heat to destroy tumor cells. The need for those minimally invasive interventions is continuously increasing due to rising case numbers of liver tumor diseases. The advantages of thermal ablation therapies lie in the much shorter treatment time as well as faster convalescence and lower risks for infections compared with a classical resection surgery. Various technologies are in clinical use as energy sources for heat generation during the thermal ablation procedure. These include microwave ablation (MWA) [2,3], radiofrequency ablation (RFA) [4,5], laser-induced interstitial thermotherapy (LITT) [6,7], and high-intensity focused ultrasound (HIFU) [8]. MWA is a relatively new technology and has become increasingly important over the other thermal therapies described. The advantages are that microwaves can propagate through all tissue types and nonmetallic materials, including water vapor as well as dehydrated, dissected, and charred tissue produced during the ablation process. In addition, larger ablation zones at consistently higher temperatures and shorter treatment duration compared with RFA have been observed [9]. Several clinical studies have shown that MWA and classical resection have comparable recurrence rates [10]. Further, MWA could enable a significant reduction of circulating tumor cells in patients with hepatocellular carcinoma (HCC) [11].

The MWA system consists of a needlelike applicator that works as an antenna at the operating frequency, a power generator, and a low-loss coaxial cable between those devices. The applicator is typically made of a semirigid coaxial cable modified by one or more slots and/or metallic ground in order to achieve monopole or dipole radiation characteristics [12]. Commercially available MWA devices work at operating frequencies of 915 MHz or 2.45 MHz with a power typically between 60 W and 100 W for a duration of about 5 to 10 min. A major challenge of MWA treatments is the accurate localization and positioning of the active part of the applicator in the center of the tumor. The clinical routine for a correct targeting of the tumor includes imaging methods such as computer tomography (CT), ultrasound (US), and magnetic resonance imaging (MRI). Currently, US and CT are most commonly used during ablation due to their widespread availability and relatively low cost. However, both modalities have limitations, these are low overview and image quality in US and poor soft tissue contrast in CT, especially without the use of contrast agents.

To date, data on the performance and duration for ablation therapies are summarized in tables from the manufacturers of the respective probes and are based on clinical studies of ex vivo and/or in vivo experiments. These data are not standardized and do not take into account patient-specific characteristics that are important for optimal ablation performance [13]. Thus, the size of the ablation zone is highly dependent on the individual patients physical constitution, perfusion, and metabolism. It follows that the success of MWA is highly dependent on the experience of the treating professional. Therefore, the demand for improved monitoring of the treatment is high and is being addressed accordingly in research. The use of microwaves as a treatment monitoring tool promises a fast and noninvasive way to observe temperature changes during the MWA. In [14], the potential of microwave tomography (MWT) as a thermal ablation monitoring tool was evaluated in silico. Further, a proof-of-concept of this principal was accordingly evaluated by experiments with ex vivo liver tissue [15]. The study showed the general feasibility of MWT as a real-time monitoring tool for MWA. A further approach that incorporated the dielectric differences between tumor, normal, and ablated tissue was given in [16]. The method is based on the evaluation of the broadband reflection signal of the microwave applicator itself in the time domain. At a boundary between tumorous and normal tissue, the dielectric discontinuity leads to a partial reflection of the electromagnetic wave propagating, from which the distance between the applicator and the tumor–normal tissue boundary can be calculated. This method requires a priori knowledge of the dielectric properties of the tissue surrounding the applicator. Therefore, a method was presented by the same group to obtain a priori knowledge by establishing a rational function between the admittance of the applicator depending on the surrounding complex permittivity of the tissue [17]. In [18], a dual-mode microwave applicator was proposed that incorporated a microwave sensing mode within the applicator itself to detect relative permittivity changes before and during the MWA procedure. It was designed as complementary to the MRI to realize a multimodal imaging for improving the success rate of current MWA systems. The dual-mode concept is based on a calibration that must be performed before the intervention.

The use of computer-assisted numerical methods is an important component for the development of novel MWA applicators and optimization of existing structures. Experiments, even on ex vivo tissues, are extremely resource- and time-intensive, and for in vivo experiments, there are ethical considerations that complicate the establishment of experimental protocols. For these reasons, the use of numerical simulation methods is particularly suitable for the investigation of the applicators performance. In [19], a Bayesian variable-number sample path optimization algorithm was described for the design of the floating sleeve antenna that was previously introduced in [20]. The optimization goal was to achieve a robust design of this antenna type that is less prone to varying dielectric properties of the surrounding tissue and, further, creates a preferably large and spherical ablation zone. A parametric design analysis based on numerical simulations of a dual-slot microwave applicator was proposed in [21]. The most optimal antenna design was defined by evaluating the reflection coefficient of the antenna and aspect ratio of the ablation zone. Further, the most optimal design was characterized with simulations and measurements.

In this work, an optimization procedure based on a parametric analysis for the design of a dual-mode microwave ablation applicator is proposed. The optimization goals are the minimal reflection coefficient of the antenna at the operating frequency as well as the maximum volume and sphericity of the ablation zone. The optimization is constrained by the microwave sensing mode to enable the detection of a tumor within the host tissue. Previous studies of the dual-mode applicator were designed by optimizing the antenna efficiency only [18]. In this paper, the simulation models as well as the objectives and constraints for the multiobjective optimization are described. Then, the results of the parametric analysis are presented and discussed.

## 2. Materials and Methods

The numerical optimization of the dual-mode microwave ablation applicator was conducted with CST Studio Suite (Dassault Systemes). The software comprises the opportunity of full-wave electromagnetic–thermal coupled simulations. Further, biological materials can be implemented in the simulation model. In this section, the simulation model of the open-ended coaxial slot applicator, as described in [18], with an operating frequency of 5.8 GHz is presented. The use of higher frequencies enables the design of smaller antennas resulting in more spherical-shaped ablation zones [22]. The parametric analysis of the applicator is described and the sensitivity requirements for the microwave sensing mode are derived. Finally, the objectives and the optimization method are presented.

### 2.1. Simulation Model

The development of accurate simulation models to determine the microwave ablation characteristics and sensitivity are essential for the numerical optimization of the dual-mode ablation applicator. CST Studio Suite offers different modules for a precise three-dimensional simulation of the applicator embedded in so called biological materials. These materials can be defined by their mechanical, dielectric, and thermal properties. The multiphysics electromagnetic–thermal coupled simulation model includes the numerical calculation of dielectric losses in tissue based on the electromagnetic field distribution according to Maxwell’s equations. The spatial distribution of the resulting losses is then transferred into distributed temperature sources for the thermal simulation model. The generated heat is determined by the so-called bioheat equation introduced by Pennes [23].

#### Parametric Analysis

**Figure 1:**
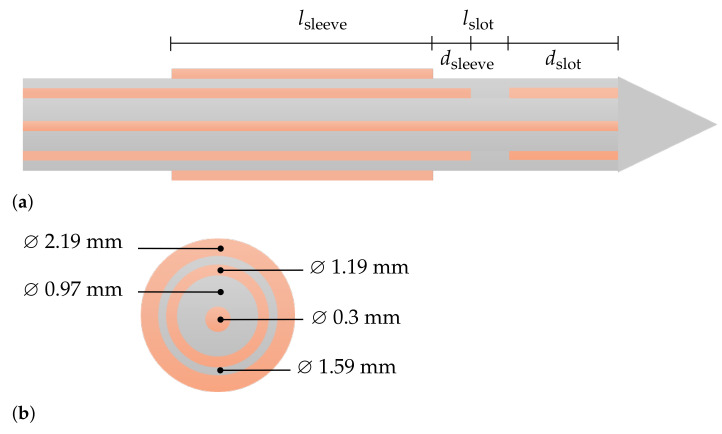
(**a**) Structure of the open-ended coaxial slot antenna with design parameter $l_{\mathrm{sleeve}}$, $d_{\mathrm{sleeve}}$, $l_{\mathrm{slot}}$, and $d_{\mathrm{slot}}$; (**b**) cross-section of the coaxial cable with a diameter of 1.19 mm surrounded by a Teflen isolation layer and a floating sleeve. Dielectric material is colored in gray and metallics are orange.

The structure of the dual-mode applicator is based on a coaxial slot antenna with an open end, as shown in Figure 1. The aim of the applicator design is to achieve efficient absorption of the energy in the surrounding biological material while preventing reverse currents on the outside of the applicator. Therefore, a sleeve as described in [20] is added to the geometry. The open-ended slot applicator is made out of a nonmagnetic coaxial cable with an outer diameter of 1.19 mm. The inner conductor and dielectric insulation between inner and outer conductor have a diameter of 0.3 mm and 0.97 mm, respectively. The applicator is covered with a PTFE-layer of 0.2 mm-thickness. Attached to this layer, the sleeve is placed with a distinct distance to the slot $d_{\mathrm{sleeve}}$ and length $l_{\mathrm{sleeve}}$. The resulting design parameters for the optimization are the distance between the distal end of the applicator and the slot $d_{\mathrm{slot}}$, the length of the slot $l_{\mathrm{slot}}$, the distance between slot and sleeve $d_{\mathrm{sleeve}}$, and the length of the sleeve $l_{\mathrm{sleeve}}$. Those parameters have a significant influence on the operating frequency, matching of the antenna, and mitigation of reverse currents. In turn, these properties affect the thermal ablation characteristics that are the volume and sphericity of the lesion as well as the sensitivity to extract the dielectric properties of the surrounding medium.

**Figure 2:**
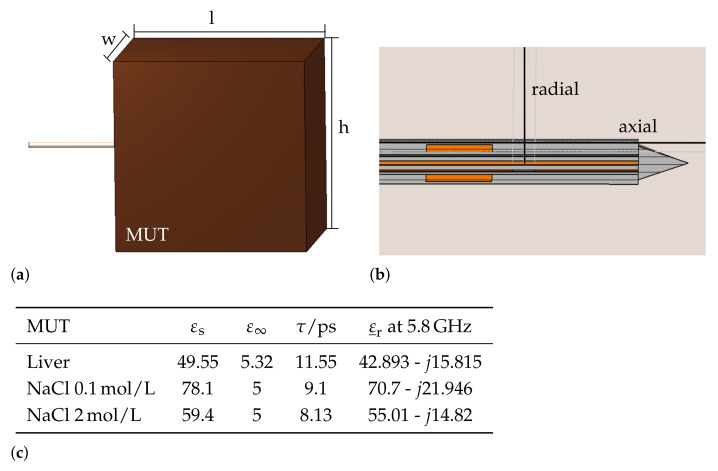
Simulation model of the dual-mode applicator. (**a**) Overview of the three-dimensional simulations model, when the applicator is inserted into MUT; (**b**) applicator geometry with evaluation curves along the axial and radial direction; (**c**) Debye model parameters of MUTs and complex relative dielectric constant at 5.8 GHz. The dielectric propertiey data of liver tissue and NaCl solutions were taken from [24] and [25], respectively.

In this study, unidirecional electromagnetic–thermal coupled simulations were performed to obtain information about the antenna efficiency and ablation zone characteristics. For the simulations, the applicator was inserted into a material under test (MUT) block, depicted in Figure 2a. Electromagnetic simulations were conducted in the frequency range from 0 GHz to 10 GHz with the transient solver. The applicator was excited by a waveguide port in order to stimulate the first propagation mode within the coaxial cable. The width w, height h, and length l of the MUT were chosen to be large enough to ensure that the electromagnetic field was either reflected or absorbed within the MUT. Thermal simulations were conducted with the thermal steady-state solver. The steady-state solver enables fast calculation of the temperature distribution with a constant input temperature to analyze and compare the thermal behavior of each applicator geometry of the parametric analysis. The temperatures were extracted along the axial and radial direction of the applicator, as shown in Figure 2b. Further, electromagnetic simulations of the applicator inserted into three reference materials as calibration standards for the microwave sensing mode were conducted. As reference materials, sodium chloride (NaCl) solutions with varying concentrations and air were selected. The dielectric properties of materials containing water, such as biological tissue and sodium chloride solutions, can be described by a complex relative permittivity $\underline{\varepsilon_{r}}=\varepsilon_{r}^{\prime }-j\varepsilon_{r}^{\prime \prime }.$ The real part of the relative permittivity corresponds to the polarizability and the imaginary part is a measure of the dielectric losses of a material. The so-called dipolar polarization is a rather slow process, which can be described mathematically by the first-order Debye equation

(1) $${\underline{\varepsilon }}_{r}=\varepsilon_{\infty }+\frac{\varepsilon_{s}-\varepsilon_{\infty }}{1+\omega \tau },$$

where $\varepsilon_{\infty }$ and $\varepsilon_{s}$ describe the polarizability at very high and static frequencies, τ is the duration after which the polarization of the material reaches the final value, and ω describes the angular frequency. In the proposed simulation model, Debye materials were implemented for the MUT with corresponding material parameters, summarized in Figure 2c. Further, the complex permittivity values from the MUTs at the operating frequency of the dual-mode applicator at 5.8 GHz are given.

As a starting point for parametric analysis of the applicator geometry, an initial design parameter set was determined via an implemented optimization tool in CST. The optimization goal was to maximize the absolute value of the reflection coefficient $|S_{11}|$ at the operating frequency of 5.8 GHz via a trust region framework when the applicator was inserted into a liver tissue block. In order to find an optimal design parameter set in terms of maximum volume and sphericity of the ablation zone, antenna efficiency, and sensitivity of the microwave sensing mode, further parametric analysis of the applicator geometry was performed. Based on the initial design parameter set, reasonable ranges of each design parameter for the parametric analysis were selected with a step width around 0.5 mm according to the in-house fabrication tolerances. The initial parameter set and sweep regions are summarized in Table 1.

**Figure 3:**
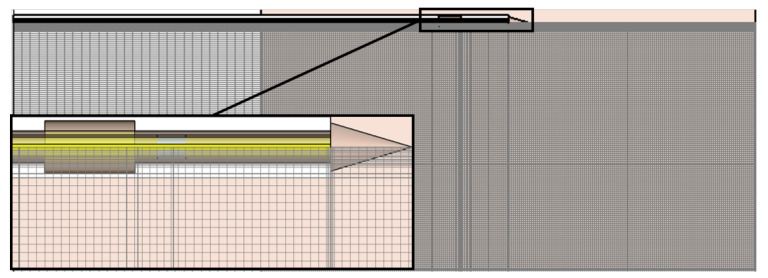
Drawing of the mesh of the simulated region. Local mesh properties were set to the applicator components to resolve small dimensions within the relatively large simulation areas.

The boundaries of the simulated region were set to open (add space) for electromagnetic and thermal simulations. Further, symmetry planes of the rotational-symmetric model were set in order to reduce the simulation time. The mesh settings of the hexahedral mesh were adaptively set until the energy within the simulation cell was converged. The resulting global mesh settings with a maximum cell side of 12/λ, where λ is the smallest wavelength of the simulation. Further, local mesh properties were applied to the relatively small components of the simulation model—the coaxial cable, the slot, the Teflon cover, and the tip with a mutual edge refinement factor of 38. A drawing of the resulting mesh of the simulated region shows the very fine mesh in the region of the applicator, Figure 3. The total number of mesh cells was around 5.7 million.

### 2.2. Sensitivity Analysis

The sensitivity of the dual-mode applicator was evaluated by means of the numerical accuracy of the relative permittivity extraction at the operating frequency of 5.8 GHz. The permittivity extraction method applied to the dual-mode applicator was described in [18]. It is based on well-known permittivity measurement techniques with an open-ended coaxial probe [26]. The relation between the simulated reflection coefficient $S_{11}$ and the complex relative permittivity of the MUT ${\underline{\varepsilon }}_{r}$ is bilinear and can be formulated as

(2) $$\begin{array}{c} {\underline{\varepsilon }}_{r}=\frac{\underline{a}\;\cdot \;S_{11}+\underline{b}}{\underline{c}\;\cdot \;S_{11}+1}. \end{array}$$

The complex coefficients $\underline{a},\underline{b},$ and $\underline{c}$ can be determined by simulations of the reflection coefficient $S_{11}$ when the applicator is inserted in three reference materials with known permittivity, ${\underline{\varepsilon }}_{r,1}$, ${\underline{\varepsilon }}_{r,2}$, and ${\underline{\varepsilon }}_{r,3}$ by following equation

(3) $$\begin{array}{c} \left(\begin{array}{c} \underline{a}\\ \underline{b}\\ \underline{c} \end{array}\right)={\left(\begin{array}{ccc} S_{11,1} & 1 & -{\underline{\varepsilon }}_{r,1}\;\cdot \;S_{11,1}\\ S_{11,2} & 1 & -{\underline{\varepsilon }}_{r,2}\;\cdot \;S_{11,2}\\ S_{11,3} & 1 & -{\underline{\varepsilon }}_{r,3}\;\cdot \;S_{11,3} \end{array}\right)}^{-1}\;\cdot \;\left(\begin{array}{c} {\underline{\varepsilon }}_{r,1}\\ {\underline{\varepsilon }}_{r,2}\\ {\underline{\varepsilon }}_{r,3} \end{array}\right). \end{array}$$

As reference materials, sodium chloride solutions with NaCl concentration of 0.1 mol/L and 2 mol/L as well as air were selected. These materials are easy to fabricate and reproduce for the NaCl solutions and well-available in hospitals as future places of action. Electromagnetic simulations for all parameter sets of the applicator inserted into each reference material were conducted and the calibration parameters for each parameter set $\underline{a},\underline{b},$ and $\underline{c}$ were calculated according to Equation (3) at the operation frequency of 5.8 GHz. In the next step, the relative permittivity of a MUT was extracted. In this study, liver tissue was selected as MUT. Hence, the sensitivity analysis was performed by comparing the simulated and the reconstructed complex permittivity of liver tissue. The sensitivity analysis method for the dielectric characterization of open-ended dielectric probe techniques was described in [27] and transferred to this work. The relative difference between extracted permittivity and the actual permittivity was considered as a measure of the extraction accuracy and determined as follows:

(4) $$\begin{array}{c} \Delta_{\mathrm{real}}=\frac{\varepsilon_{\mathrm{ext}}^{\prime }-\varepsilon_{\mathrm{MUT}}^{\prime }}{\varepsilon_{\mathrm{MUT}}^{\prime }} \end{array}$$

(5) $$\begin{array}{c} \Delta_{\mathrm{imag}}=\frac{\varepsilon_{\mathrm{ext}}^{\prime \prime }-\varepsilon_{\mathrm{MUT}}^{\prime \prime }}{\varepsilon_{\mathrm{MUT}}^{\prime \prime }} \end{array}$$

The dielectric contrast between tumor and healthy liver tissue varies per patient [28,29]. For the detection of a relative dielectric contrast in the range of 20%, the extraction error shall not exceed 10% in order to ensure the functionality of the dual-mode applicator. This means that parameter sets within the parametric analysis with a higher extraction error cannot be considered as optimum for the following optimization.

### 2.3. Objectives and Optimization Method

In this study, the efficiency of the applicator and the ablation zone characteristics in terms of shape and size were optimized under constraints of the applicator’s sensitivity. In the following, the objectives of the optimization are described.

#### 2.3.1. Applicator Efficiency

The applicator efficiency is related to the computed reflection coefficient $S_{11}$ in dB at the operating frequency at 5.8 GHz. The higher $|S_{11}|$, the more energy is absorbed into the surrounding material. The reflection coefficients of all design parameter combinations were calculated, normalized to 50 Ω and exported to MATLAB (Mathworks Inc. Natick, MA, USA) for further postprocessing steps.

#### 2.3.2. Volume and Sphericity of Ablation Zone

It is desirable to achieve preferably large and spherical ablation zones. These properties can be described by the two objective metrics—volume and sphericity of the ablation zone. The heating pattern of each design parameter combination of the applicator inserted into liver tissue was simulated according to loss calculations from the electromagnetic simulations. The input power was set to 10 W at the operating frequency of 5.8 GHz. The thermal properties of liver tissue were set to a thermal conductivity of 0.469 W/K/m. Further, bioheat specific properties were considered by a blood-flow coefficient of 68000 W/K/$m^{3}$ and a basal metabolic rate of 1200 W/$m^{3}$. The resulting temperatures were then extracted along the axial and radial dimension of the applicator. The spatial resolution of the temperature extraction along the curves was 0.1 mm. It was assumed that temperatures above 55 °C result in tissue necrosis; therefore, we determined the axial (long axis, LA) and radial (short axis, SA) extension of the ablation zone. The volume and sphericity of the ablation zone were then calculated for each design parameter combination by

(6) $$\begin{array}{c} V=\frac{4}{3}\pi \;\cdot \;\mathrm{LA}\;\cdot \;{\mathrm{SA}}^{2} \end{array}$$

(7) $$\begin{array}{c} S=\frac{2\mathrm{SA}}{\mathrm{LA}}. \end{array}$$

#### 2.3.3. Multiobjective Optimization

The optimization of the dual-mode applicator performance comprises multiple object functions $f_{i}$, with i=1,2,3, each with a distinct optimization goal. The objective functions relate to the antenna efficiency, the volume, and the sphericity of the ablation zone. The overall optimization goal was to trade-off these objectives in order to find an overall solution. The multiobjective optimization problem can be defined as

(8) $$\begin{array}{c} \mathrm{maximize}:\;f\left(x\right)=[f_{1}\left(x\right),f_{2}\left(x\right),f_{3}\left(x\right)] \end{array}$$

$$\begin{array}{c} \mathrm{with}\;f_{1}\left(x\right)=\left|S_{11}\left(x\right)\right|,\\ f_{2}\left(x\right)=V\left(x\right),\\ f_{3}\left(x\right)=S\left(x\right), \end{array}$$

with x= $x_{1},x_{2},x_{3},\;\mathrm{and}\;x_{4}$∈X, where X contains all parameter sets of the design parameters. The objective function $f_{i}$, with i=1,2,3, represents the objective metrics that were introduced above. The goal of this study is to find a parameter set $\hat{x}$ that is Pareto-optimal in the sense that no other parameter set x exists such that $f\left(x\right)\geq f\left(\hat{x}\right)$ [30].

## 3. Results

The parametric analysis includes 225 permutations of the applicator geometry. Each electromagnetic simulation required approximately 25 min ± 5 min, and thermal simulations took approximately 1 min ± 0.5 min. All simulations were performed remotely on a CST solver server with 2 × 12 cores @ 2.99 GHz. In the following, the Pareto-optimal solution of the parametric analysis for the optimization of the dual-mode applicator geometry is derived. The corresponding characterization of the Pareto-optimal applicator geometries are presented in terms of the applicator efficiency and ablation zone size and shape.

### 3.1. Pareto-Optimal Parameter Sets

**Figure 4:**
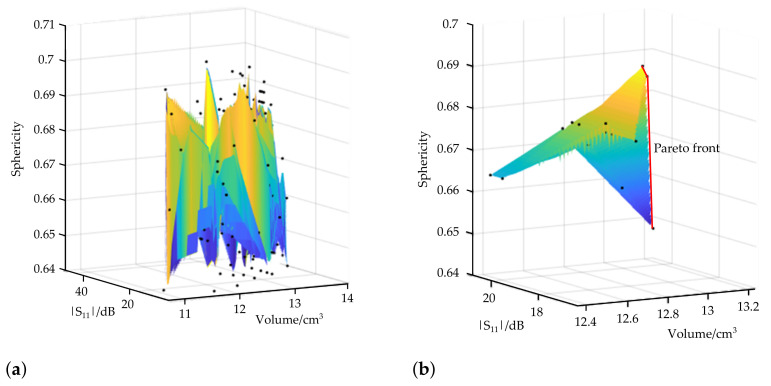
Three-dimensional Pareto plot of (**a**) all parametric results and (**b**) those under constraints of the sensitivity requirement of the dual-mode applicator.

The Pareto-optimal solution of the parametric analysis can be graphically determined. The values of each objective function $f_{i}$, with i=1,2,3, are applied to the three-dimensional Pareto plot, Figure 4. The number of possible Pareto-optimal solutions decrease significantly by considering the sensitivity requirement from the initial 225 parameter sets (Figure 4a) to 15 possible parameter sets (Figure 4b). The so-called Pareto front describes the set of Pareto-optimal solutions for the case that no change of a certain objective could lead to an overall improvement of the objective function. It is drawn as a red line in Figure 4b. In this study, three parameter sets lie on the Pareto front. These sets are further referred to as Pareto-optimal parameter sets. The corresponding dimensions of each design parameter, objectives and sensitivity, are summarized in Table 2. It is remarkable that only the length of the sleeve varies for the resulting Pareto-optimal parameter sets. The reflection coefficient and ablation zone characteristics were determined by simulations when the applicator was inserted into liver tissue.

### 3.2. Applicator Characterization

**Figure 5:**
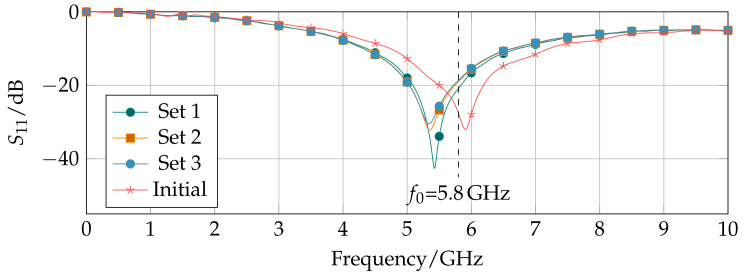
Reflection coefficient of three Pareto-optimal parameter sets for the dual-mode applicator inserted into liver tissue.

The simulated reflection coefficients from 0 GHz to 10 GHz of the Pareto-optimal dual-mode applicator designs inserted into a liver tissue block show a good matching at the operating frequency of $f_{0}$= 5.8 GHz below −15 dB (Figure 5). The Pareto-optimal parameter sets are very similar. Only one of four design parameters varies in the length of the sleeve $l_{\mathrm{sleeve}}$. With decreasing sleeve length, the reflection further decreases. In comparison with the optimized applicator design, the reflection coefficient of the initial design parameter set is shown. The initial parameter set was computed by an optimization of the reflection coefficient at the operating frequency. As a result, the corresponding reflection coefficient is $S_{11,\mathrm{initial}}$ = −29.8 dB compared with $S_{11,1}$= −18.61 dB, $S_{11,2}$= −18.97 dB, and $S_{11,3}$= −20.77 dB for parameter sets 1, 2, and 3, respectively. The initial parameter set presents a better matching of the applicator to liver tissue. However, the extracted error $\Delta_{\mathrm{initial}}$ = 15.83% is contrastingly higher compared with the Pareto-optimal designs and exceeds the given sensitivity requirement.

**Figure 6:**
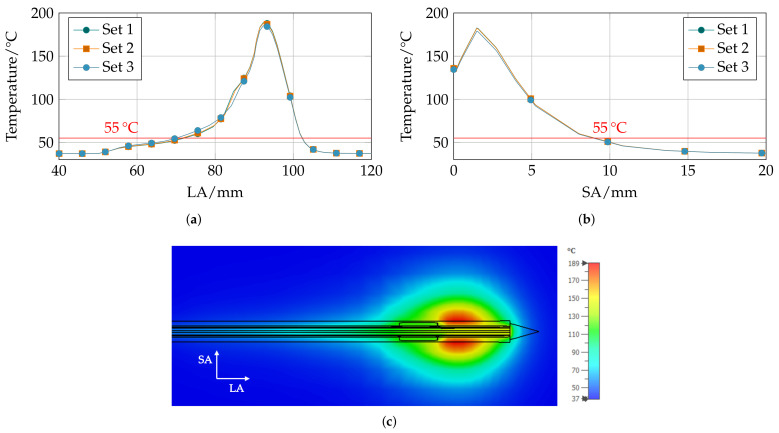
Thermal simulation result of Pareto-optimal parameter sets. Temperature along the (**a**) axial (LA) and (**b**) radial (SA) direction for all three sets and (**c**) the thermal ablation zone from three-dimensional simulations of parameter set 1.

Regarding the results of the corresponding thermal simulations, the axial temperature distribution varies slightly between the parameter sets. With increasing sleeve length, the sphericity of the ablation zone increases and the volume decreases; whereas, the radial temperature distribution practically shows no differences between the three parameter sets. The resulting temperature distributions along the axial (LA) and radial (SA) direction are shown in Figure 6a,b and the heat map of the temperature distribution from the three-dimensional simulation model is given in Figure 6c, exemplary of design parameter set 1.

## 4. Discussion

In this work, a parametric analysis of a dual-mode microwave applicator was conducted with the aim to optimize the applicator’s performance in terms of efficiency as well as the size and shape of the ablation zone. Moreover, the dual-mode functionality was considered in the optimization by constraining the simulation results. In the past, many MWA applicators have been proposed in literature that were designed by matching the antenna to healthy liver tissue properties as surrounding. For a comprehensive optimization of the applicator design parameters, a multiphysical approach must be applied that incorporates electromagnetic–thermal coupled simulations. The starting point of the optimization was selected by an optimization of the applicator efficiency only within liver tissue. The following multiobjective optimization under constraints of the sensitivity resulted in different applicator geometries.

This study presents a multiobjective optimization procedure based on numerical simulations of the dual-mode applicator inserted into liver tissue as MUT. The sensitivity investigations are a key factor of the optimization procedure as it is a fundamental property to ensure a proper dual-mode functionality. The sensitivity was defined as the error between the extracted permittivity from the simulated reflection coefficient and the actual reference permittivity. Therefore, electromagnetic simulations of the applicator designs inserted into three reference materials were conducted. By doing so, the applicator performance inserted in materials with different dielectric properties to liver tissue was considered. Regarding applicator-matching of the Pareto-optimal design parameter, the optimal matching frequencies of the antenna were shifted compared with the operating frequency of 5.8 GHz; although, the matching of at least −15 dB is still good enough to create large lesions. The sensitivity analysis can be seen as a suitable method to achieve an applicator design that is not so prone to permittivity changes in the surrounding area.

From the resulting Pareto-optimal design parameter sets, a certain optimal design of the applicator with a distance between tip and slot of $d_{\mathrm{slot}}$ = 7.7 mm, a slot length of $l_{\mathrm{slot}}$ = 1.75 mm, and a distance between slot and sleeve of $d_{\mathrm{sleeve}}$ = 0.55 mm was achieved. From the optimization results, it can be derived that the design is rather insensitive to changes of the sleeve length $l_{\mathrm{sleeve}}$. A possible explanation for this phenomenon is the different geometry of the open-ended slot structure compared with other MWA applicator designs reported in literature. The proposed applicator does form a standing wave within the coaxial cable with an electric field minimum and a current density maximum at the position of the slot. That makes it less prone to reverse currents along the outer conductor of the coaxial cable.

This study is limited due to the sole use of numerical solutions to derive the electromagnetic and thermal properties of the microwave applicator; therefore, the calculated extraction errors include numerical uncertainties. The next step will be the experimental verification of the proposed applicator geometries.

## Figures and Tables

**Table 1:** Initial value and sweep range of design parameters for optimization of the dual-mode applicator.

Design Parameter	Initial Value/mm	Sweep Range	Relative Change
Length sleeve $l_{\mathrm{sleeve}}$	4.4	3.52, ..., 5.28	±20%
Distance sleeve $d_{\mathrm{sleeve}}$	1.1	0.55, ..., 1.65	±50%
Length slot $l_{\mathrm{slot}}$	1.4	1.05, ..., 1.75	±50%
Distance slot $d_{\mathrm{slot}}$	7.1	5.9, ..., 8.3	±18%

**Table 2:** Design parameter and objective values from Pareto-optimal parametric sets.

	Design Parameter/mm				Objectives			Sensitivity
Set	$l_{\mathrm{sleeve}}$	$d_{\mathrm{sleeve}}$	$l_{\mathrm{slot}}$	$d_{\mathrm{slot}}$	$|S_{11}|$/dB	*V*/cm^3^	Sphericity	$\Delta_{\mathrm{real}}$
1	5.28	0.55	1.75	7.7	18.61	12.94	0.6908	2.04%
2	4.84	0.55	1.75	7.7	18.97	13	0.6874	5.02%
3	3.52	0.55	1.75	7.7	20.77	13.24	0.648	5.01%

## Data Availability

The data presented in this study are available on request from the corresponding author.

## Abbreviations

The following abbreviations are used in this manuscript:

MDPI	Multidisciplinary Digital Publishing Institute
RFA	Radiofrequency ablation
MWA	Microwave ablation
LITT	Laser-induced thermo-therapy
HIFU	High-intensity focused ultrasound
HCC	Hepatocellular carcinoma
CT	Computer tomography
US	Ultrasound
MRI	Magnetic resonance imaging
MWT	Microwave tomography
MUT	Matrial under test
NaCl	Sodium Chloride
